# Mid-Term Outcomes of a Rectangular Stem Design with Metadiaphyseal Fixation and a 135° Neck–Shaft Angle in Reverse Total Shoulder Arthroplasty

**DOI:** 10.3390/jcm14020546

**Published:** 2025-01-16

**Authors:** Yacine Ameziane, Laurent Audigé, Christian Schoch, Matthias Flury, Hans-Kaspar Schwyzer, Alessandra Scaini, Emanuele Maggini, Philipp Moroder

**Affiliations:** 1 Shoulder and Elbow Surgery, Schulthess Clinic Zürich, 8008 Zürich, Switzerland; 2 Orthopedic Practise Clinic Muenster, 48143 Muenster, Germany; 3 Research and Development, Schulthess Clinic Zürich, 8008 Zürich, Switzerland; 4 Surgical Outcome Research Center, Department Clinical Research, University Hospital Basel, 4031 Basel, Switzerland; 5 St. Vinzenz Clinic, 87459 Pfronten, Germany; 6 Centrum for Orthopedics and Neurosurgery, 8304 Wallisellen, Switzerland

**Keywords:** shoulder, reverse total shoulder arthroplasty, humeral stem fixation, inclination

## Abstract

**Background/Objectives**: Classical reverse shoulder arthroplasty (RSA) with a high neck–shaft angle (NSA) of 155° has shown satisfactory outcomes. However, newer RSA designs aim to improve results by modifying the stem design. This study evaluates the 5-year outcomes of a stem design featuring a rectangular metadiaphyseal fixation and a 135° NSA. **Methods**: This prospective bicentric case series included and longitudinally followed up patients that were treated for cuff arthropathy, massive irreparable rotator cuff tears, or eccentric osteoarthritis using a non-cemented rectangular metadiaphyseal fixation stem with a 135° NSA (Univers Revers, Arthrex, Naples, FL, USA). Subjective and objective functional outcome scores (Constant–Murley Score (CS), Shoulder Pain and Disability Index (SPADI), and Subjective Shoulder Value (SSV)), range of motion (ROM), radiographic outcome, adverse events, complications, and quality of life were investigated. **Results**: This study enrolled 132 patients (59% female, mean age 75 years, SD 6). At the 5-year follow-up, subjective and objective outcomes significantly improved compared to baseline: CS (32.9 to 71.7, *p* < 0.001), SPADI (38.7 to 86.2, *p* < 0.001), and SSV (43.0 to 84.1, *p* < 0.001). ROM improved in flexion (80° to 142.4°, *p* < 0.001), abduction (71.5° to 130.2°, *p* < 0.001), internal rotation (*p* < 0.001), internal rotation at 90° abduction (12.7° to 45.0°, *p* < 0.001), and abduction strength (0.8 kg to 5.2 kg, *p* < 0.001). External rotation remained unchanged (32.1° to 32.0°, *p* = 0.125), but external rotation at 90° abduction improved (20.9° to 52.7°, *p* < 0.001). No signs of implant migration, subsidence, shift, tilt, alignment loss, or wear were observed, but scapular bone spur formation (11%), scapular notching grade 1 (10%), bone resorption (10%), and partial humeral radiolucent lines (1%) were reported. **Conclusions**: Rectangular stems with metadiaphyseal fixation and a 135° neck–shaft angle in RSA consistently improve shoulder function, showing no aseptic loosening and minimal radiological changes at 5 years.

## 1. Introduction

Reverse total shoulder arthroplasty (RTSA) is a frequently used and established procedure for the treatment of severe shoulder pathologies like cuff tear arthropathy (CTA), eccentric osteoarthritis, failed anatomic shoulder replacement, and proximal humerus fractures. Several studies presented promising mid- to long-term results of the classical medializing and distalizing Grammont design prosthesis with a 155° inclination angle [1,2,3].

Despite encouraging survival rates, the initial Grammont design may lead to various postoperative problems and complications like postoperative instability, infection, scapular notching, acromial or scapular fractures, or implant loosening [4,5,6]. While some of these manifest during short-term follow-up after surgery, others increase in occurrence only during mid- to long-term evaluation [7,8].

To reduce mechanical complications and optimize functional results, the design of the RSA has been continuously refined. The reduction of the humeral inclination from 155° to a lateralizing 135° design was adopted to improve rotational capacity and reduce scapular notching [9,10].

However, short- to mid-term data remain contradictory regarding the postoperative range of motion (ROM). While a scientific review of 2222 shoulders revealed improved external rotation of patients treated with a 135° design, recently published prospective randomized results showed no differences between the two neck–shaft designs concerning the postoperative mobility at short-term follow-up [9,10]. In a CT-based study, Werner et al. revealed a significant increase in impingement-free ROM in favor of the 135° design compared to higher neck–shaft angles, and clinical short- to mid-term outcomes showed a statistically significant decrease in postoperative scapular notching [11]. While these CT-based results could be transferred into a clinical setting, presenting a significantly reduced rate of scapular notching in a prospective randomized data set at short-term follow-up, mid- to long-term data are still to be published [9]. Nonetheless, lowering the NSA has been hypothesized to be associated with potential complications in terms of instability [10,12].

Even though aseptic stem loosening seems to be a rare complication of RTSA, Grey et al. demonstrated an increasing prevalence during mid- to long-term follow-up examinations [8]. Potentially, modern stem designs can decrease the rate of loosening due to the reduction of scapular notching and polyethylene wear-induced osteolysis, as well as by means of improved design features facilitating reliable ingrowth without cementation.

Therefore, this prospective bicentric case series with a minimum follow-up of five years was conducted to evaluate clinical results concerning the impact of a reduction of the humeral NSA to a 135° design with additional focus on radiographic changes during mid- to long-term follow-up when using a rectangular stem with meta-diaphyseal fixation.

## 2. Materials and Methods

### 2.1. Study Design and Population

This prospective bicentric case series included patients from one Swiss and one German clinic. All patients were treated between November 2013 and December 2016 and originated from a larger multicenter cohort with strict inclusion and exclusion criteria [13]. Included patients received RSA for cuff arthropathy, massive irreparable rotator cuff tears, or eccentric osteoarthritis. Patients treated for previous ipsilateral anatomic or reverse shoulder arthroplasty, fracture sequelae, proximal humerus fractures, rheumatoid arthritis, or an active malignancy were excluded.

While 85 patients (64%) reported hypertension as a comorbidity, 33 (25%) patients revealed a history of previous cardiac diseases. Moreover, diabetes was reported in eight cases (6%), whereas overweight was noted in 33 patients (25%).

Informed consent was obtained from all patients, and institutional review board/ethics committee approval was granted by the Cantonal Ethics Committee of Zürich (KEK-ZH-Nr: 2013-0185).

### 2.2. Study Intervention

**Figure 1:**
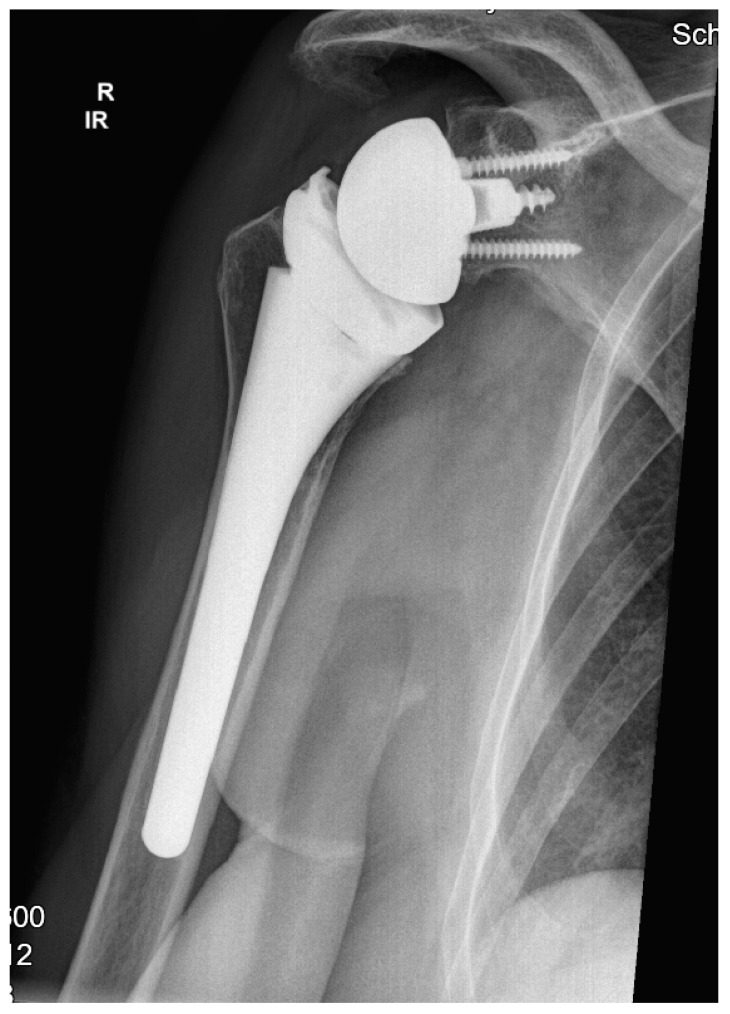
Example at the five-year follow-up after reverse shoulder arthroplasty with 135° NSA and a cementless metadiaphyseal fixation stem (Univers Revers, Arthrex, Naples, FL).

All patients were treated with RSA using the Univers Revers prosthesis with a 135° NSA (Arthrex, Naples, FL, USA; Figure 1) combined with the Universal Glenoid (Arthrex, Naples, FL, USA). The design of this prosthesis is based on the usage of a rectangular metadiaphyseal locking stem that is combined with a metaphyseal cup which be used centered or with either 2 mm left or right offset according the humeral diaphysis. This humeral inlay design is further completed by the usage of a standard or constrained +3 mm or +6 mm humeral polyethylene liner that offers adaptation to the glenosphere diameters’ different concavity depths (jump heights) from 9.8 mm (36 mm standard) to 13.9 mm (42 mm constrained) and following stability ratios of 193–194% using a standard liner as well as stability ratios of 277–300% using constrained liner designs [14].

Preoperative decision-making and surgical planning were based on clinical examinations as well as radiographs using a set of templates for glenoid and humeral component sizing. In case of suspicion of large glenoidal defects and/or humeral head subluxation, patients received preoperative computed tomography (CT) scans to assess the exact glenoidal defect size for better preoperative planning and further ensuring the accuracy of implantation.

A rectangular metadiaphyseal locking stem could be used with sufficient primary stability without cement in all 132 patients (100%). One hundred and thirty patients (98%) were treated with a +3 mm inlay liner, while a 6 mm inlay liner was used in two patients (2%). Additionally a 6 mm humeral spacer was used in one patient (1%).

A glenosphere diameter of 36 mm was used in 51 patients (39%), while glenosphere diameters of 39 mm and 42 mm were used in 57 patients (43%) and 24 patients (18%), respectively. While a neutral standard glenosphere (standard) was used in 18 patients (14%), 114 patients (86%) were treated with a 4 mm lateralized (+4 mm) glenosphere.

All patients were treated via a deltopectoral approach. Optimal implant sizing and deltoid and general joint tension were further attained through implant trials. The subscapularis tendon was reattached if a tension-free refixation was possible. While 129 (98%) subscapularis tendons were repaired, three patients (2%) presented an irreparably torn tendon.

Postoperatively, patients were treated by the institution-specific rehabilitation plans that were both based on immobilization in an abduction sling for one to four weeks with immediate passive mobilization during physical therapy. While one institution started active mobilization under direct physiotherapeutic supervision after six weeks, the second institution started active mobilization in the third postoperative week.

### 2.3. Follow-Up Outcome Parameters

Subjective and objective clinical and functional examinations were documented preoperatively (baseline), as well as, along with the assessment of adverse events (AE) and complications, at 6 weeks (only AE and quality of life), six months, one year, two years, and five years after surgery.

The Constant–Murley Score [15] (CS), the Shoulder Pain and Disability Index (SPADI) [16], and the Subjective Shoulder Value (SSV) [17] were obtained at the six month-, one-, two-, and five-year follow-ups. Either a spring balance (Pesola AG, Schindellegi, Switzerland) or an Isobex dynamometer (Cursor AG, Bern, Switzerland) was used to obtain the abduction strength. Furthermore, ROM was assessed using a standardized protocol. The Apley scratch test was used to assess the capacity of internal rotation. Quality of life was assessed using the European Quality of Life 5-Dimensions 5-Level Questionnaire [18]. During follow-up examinations patients were asked whether they would agree to undergo surgery again and to what extent their expectations of the operations were fulfilled (0 = not at all; 10 = fully).

### 2.4. Radiographic Follow-Up

All patients underwent preoperative radiographic examinations as well as magnetic resonance imaging, CT arthrography, or ultrasound examinations to assess the integrity of the rotator cuff preoperatively. Further, the grade of tendon retraction, fatty infiltration, and muscle atrophy was assessed according to previously described scores [19,20,21].

Radiographs of anterior, posterior, and axillary views were obtained at the six-month, one-, two-, and five-year follow-ups. All radiographs were reviewed, evaluated, and graded centrally and independently by two fellowship-trained shoulder surgeons concerning signs of implant loosening, subsidence, shift, tilt, displacement, loss of alignment, periprosthetic fractures, instability, implant wear or breakage, and signs of bone formation and resorption (scapular notching [22] and heterotopic ossification [23]), according to an international consensus core outcome set on radiographic monitoring of shoulder arthroplasties [24]. Further parameters like the lateralization angle (LSA), distalization angle (DSA) [25], and glenoid overhang were determined.

### 2.5. Adverse Events

Local adverse events (AEs) including surgical complications were recorded and classified according to a previously described standardized method [26,27]. All events were assessed with regard to their severity, as well as their relationship with the RSA procedure, notably to identify serious adverse events (SAEs, including revision operations) and serious adverse device effects (SADEs) [24].

### 2.6. Data Management and Statistical Analyses

Study data were managed using the REDCap (Research Electronic Data Capture) system [28] and exported for statistical analysis using Intercooled Stata version 17 (StataCorp LP, College Station, TX, USA). Baseline patient demographic, radiological, and functional parameters were tabulated using standard descriptive statistics. The change of outcome parameters from baseline (preoperative) to the 5-year follow-up was graphed and analyzed using standard linear regression analyses; generalized linear mixed models were used to account for repeated measurements when outcome data were available at each clinical follow-up examination, as applicable.

In addition, to explore factors associated with acceptable internal rotation at 5 years, preoperative baseline characteristics of patients showing Apley’s test reaching at least L3 were compared to characteristics of patients with poorer internal rotation (Apley’s test < L3) using univariable parametric statistical testing.

Adverse events occurring between the 2- and 5-year follow-up were described. The risk of implant revision was assessed by a Kaplan–Meier survival analysis. All analyses were implemented with the significance level set at 0.05.

## 3. Results

### 3.1. Demographics and Baseline Parameters

**Figure 2:**
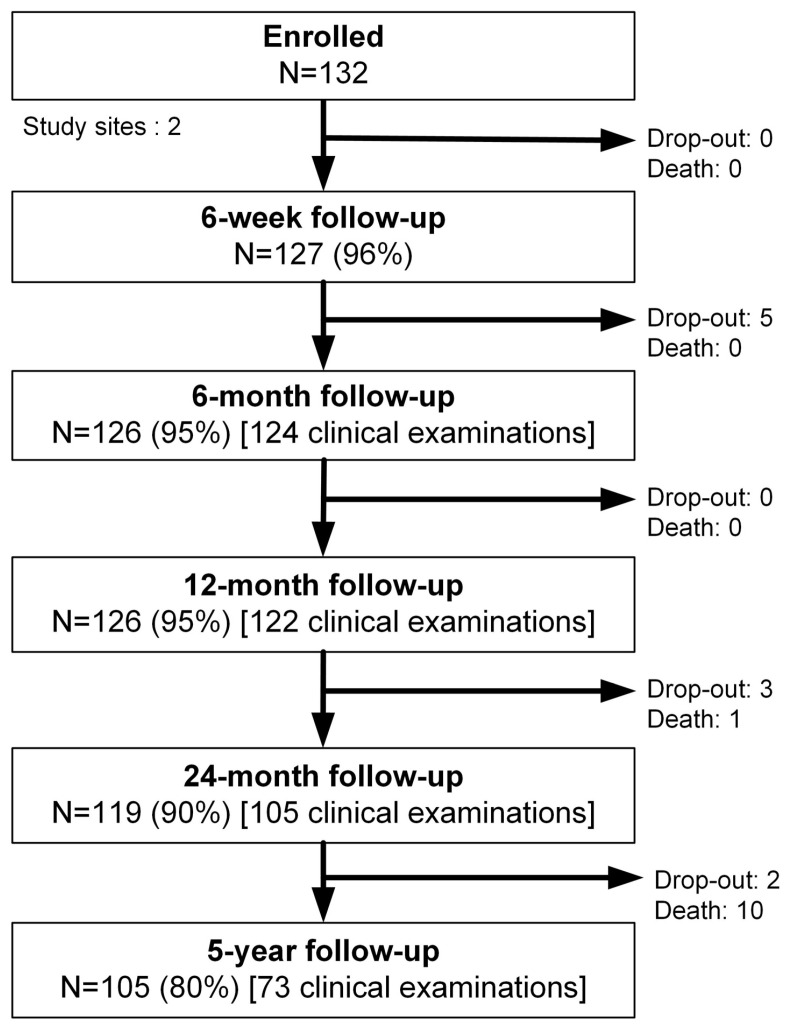
Flow chart—enrolled patients and follow-up.

During the period of inclusion, 132 patients (female: 78, 59%) with a mean age of 75 years (SD 6, range 58–91 years) could be enrolled in this study (Table 1). One hundred and eighteen patients (89%) presented comorbidities, whereas 66 patients (50%) presented a history of arthritic diseases in other joints. After 5 years, 105 patients (80%) were available for the final follow-up, of which 73 underwent clinical examination, while radiographic analysis could be performed in 70 cases. Subjective values (SPADI, SSV, quality of life indices) and AEs were postally obtained from 32 patients that were not able to present at a clinic due to the COVID-19 pandemic or poor general health condition (Figure 2). The initial CS, SPADI, and SSV were 33 points (pts.) (SD 15), 39 pts. (SD 17), and 43% pts. (SD 18), respectively. Baseline active ROM was limited to 80° (SD 34) flexion, 72° (SD 28) abduction, 32° (SD 22) external rotation, and 21° (n = 62; SD 30) external rotation at 90° abduction (Table 2). Fifty-one patients further presented a positive external rotation lag sign. Further internal rotation was limited to the lateral aspect of the thigh in 11 patients (8%), to the gluteal region in 33 patients (25%), to the lumbosacral region in 39 patients (30%), to L3 in 27 patients (21%), and to TH 12 and the interscapular region in 20 patients (15%). Internal rotation at 90° abduction was limited to 13° (SD 18).

### 3.2. Radiological Outcome

**Figure 3:**
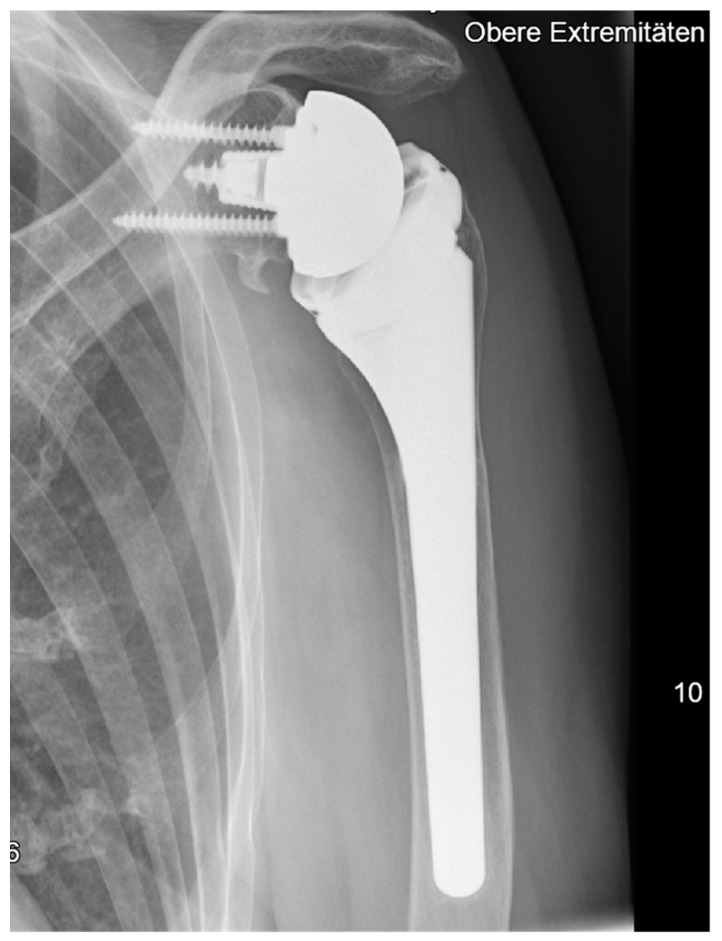
Signs of diaphyseal bone resorption at the 5-year follow-up after RTSA implantation.

**Figure 4:**
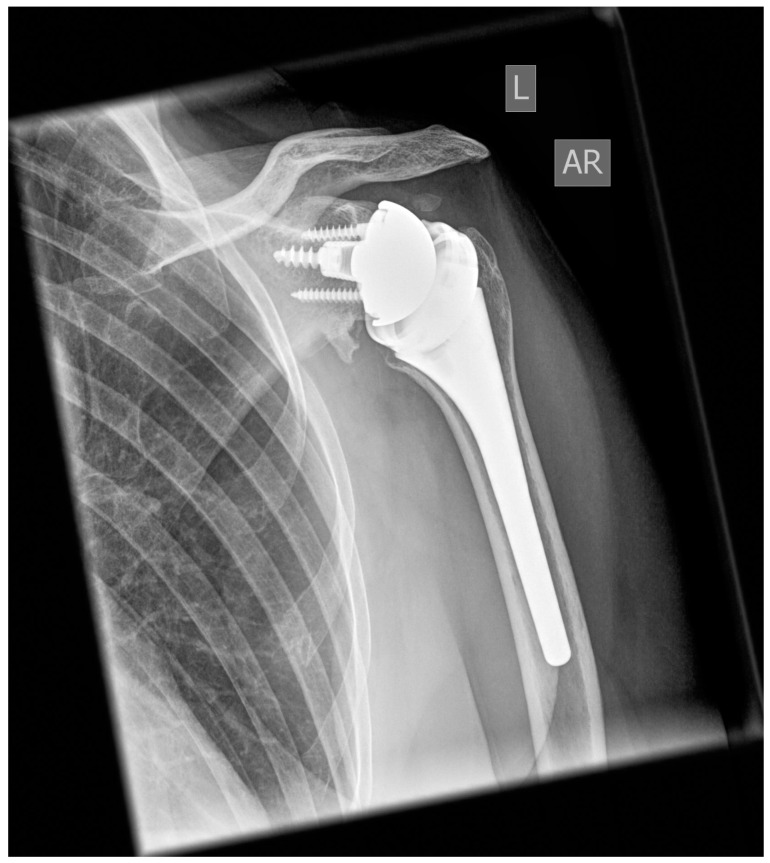
Reverse shoulder arthroplasty at the 5-year follow-up showing grade 1 scapular notching and consecutive scapular bone spur formation.

Anteroposterior radiographic imaging was available in 70 patients at the final five-year follow-up, with no signs of implant instability, malpositioning, or migration, including subsidence, tilt, or shift. One patient (1%) presented signs of grade 1a radiolucency at the humeral metaphysis. Bone resorption was found in two patients (3%) at the humeral metaphysis and in five patients (7%) at the humeral diaphysis (Figure 3). While 63 patients (90%) showed no signs of scapular notching, seven patients (10%) revealed evidence for grade 1a scapular notching during the five-year follow-up (Figure 4). Furthermore, eight patients (11%) revealed signs of scapular bone spur formation (Figure 4).

There were no postoperative humeral-sided fractures noticed at the five-year follow-up, while one acromion fracture (1%) was observed. Finally, no implant breakage or early wear was detected. The mean LSA was 86.0° (SD 10.6°), ranging from 38.0° to 105.0°, and the mean DSA was 44.4° (SD 10.9°), ranging from 3.0° to 83.0°. Concerning the glenoid component placement, radiographs showed an inferior glenosphere overhang of 3.8 mm (SD 3.5; range −4.0 to −21.0) and an inferior baseplate tilt of 4.8° (SD 4.8°; range −7.0° to 20.0°).

### 3.3. Adverse Events, Survival Rate, and Complications

**Figure 5:**
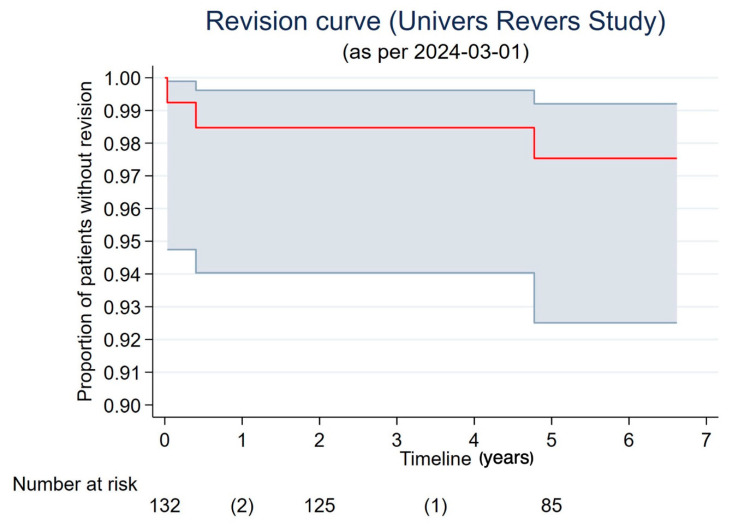
Kaplan–Meier curve: implant survival rate, including number at risk at specific time points. Numbers in parentheses indicate revisions conducted prior to the 2-year follow-up and at the 2- and 5-year follow-ups.

Short-term AEs have previously been described in a prior publication [13]. From the present cohort, two local AEs occurred during the two- to five-year follow-up period. One patient suffered persisting pain after a fall on the shoulder without radiological signs of a periprosthetic fracture or implant loosening, while another patient experienced an infection (SAE) with the need for two-stage revision. No events of instability or dislocation of the implant were noted during the follow-up period. The overall implant survival rate within the included cohort was 0.98 at the five-year follow-up (95%, CI 0.93–0.99) (Figure 5). While one patient suffered an acromion fracture (1%), seven patients (10%) showed radiological signs of scapular notching, and grade 1a humeral loosening was observed in one further patient (1%).

### 3.4. Subjective and Objective Outcomes and Quality of Life

**Figure 6:**
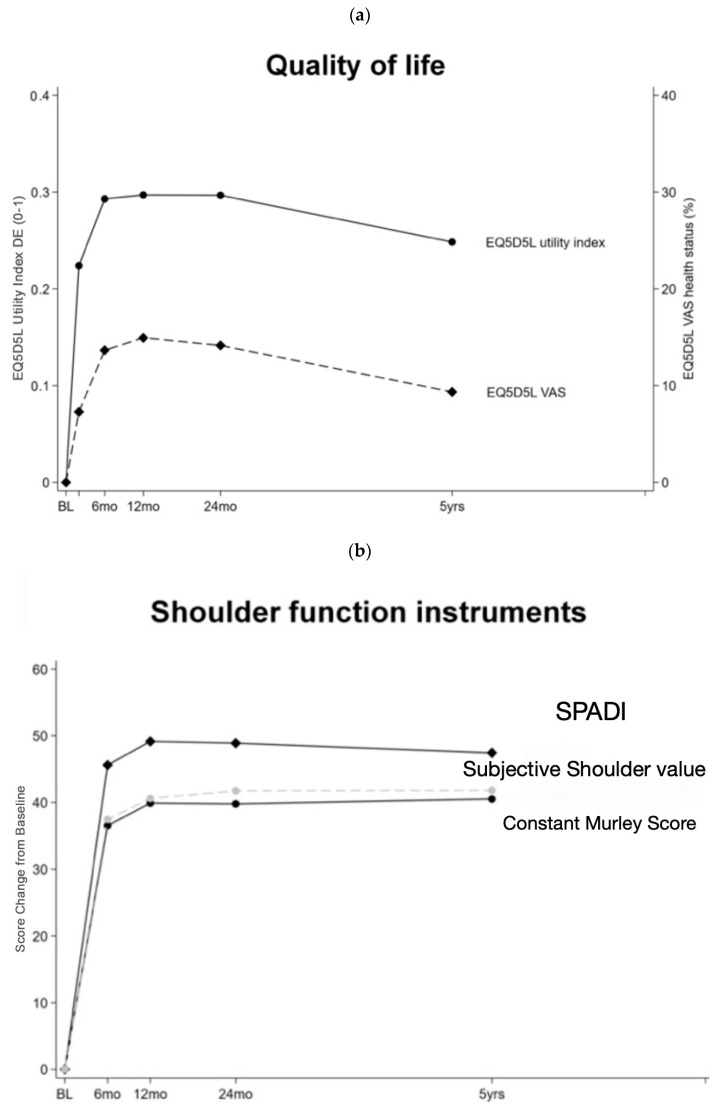

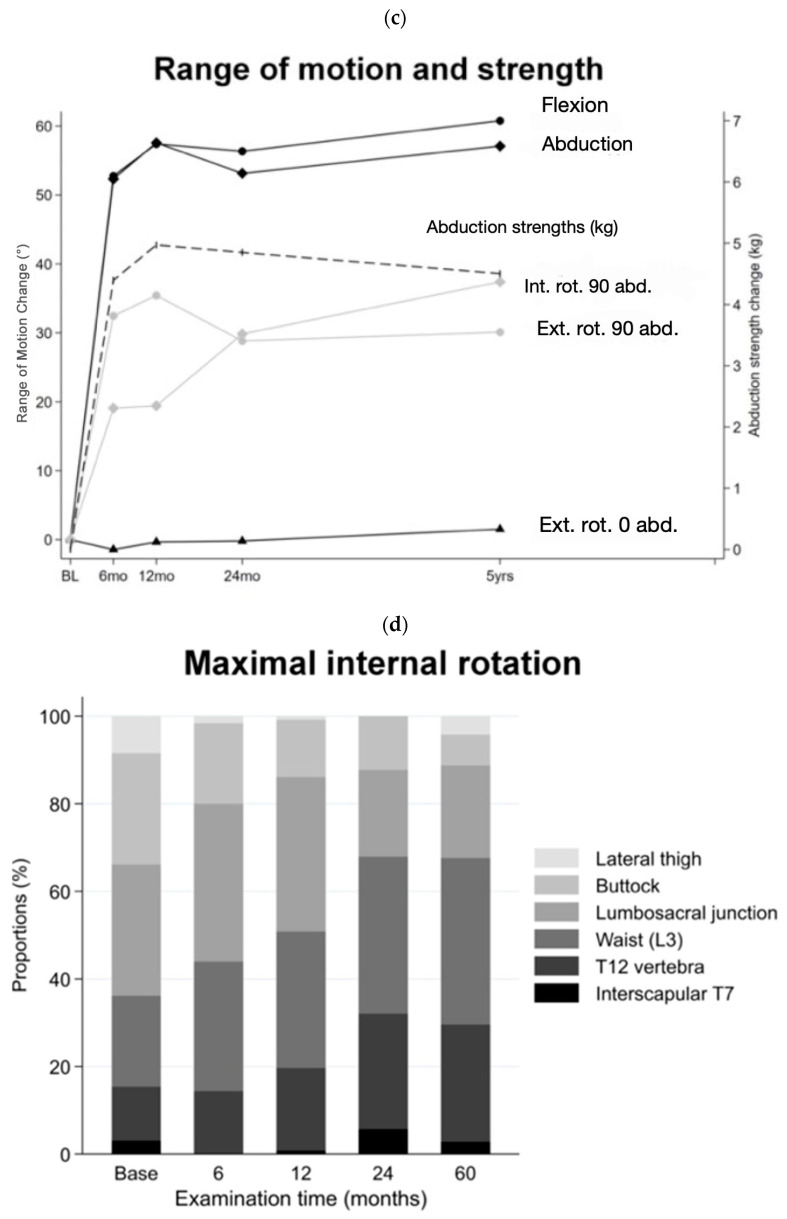
Longitudinal changes of subjective and objective outcome parameters and ROM from baseline to the five-year follow-up. (**a**) Longitudinal changes of subjective quality of life parameters from baseline to five-year follow-up. (**b**) Longitudinal changes of the SPADI, the Constant-Murley Score and the Subjective Shoulder-Value from baseline to five-year follow-up. (**c**) Longitudinal changes of ROM from baseline to five-year follow-up. (**d**) Longitudinal changes of ROM in terms of maximal internal rotation heights from baseline to five-year follow-up.

Over the course of measurements at each follow-up, the EQ5D5L Utility Index and the EQ5D5L VAS Health Status improved significantly pre- to postoperatively (Table 2, Figure 6a). Patients presented statistically significant improvements in the CS and SPADI score as well as the SSV, which was sustained over the course of five years (Figure 6b, Table 2). ROM improved in flexion, abduction, and internal rotation at 90° (Table 2, Figure 6c). External rotation showed no statistical improvement compared to the preoperative measurements, whereas external rotation at 90° abduction improved (Table 2, Figure 6c). Internal rotation improved to higher percentages of satisfactory results (Table 2, Figure 6d), and 90° abduction strength further improved over the course of follow-up to a final strength of 5.2 kg at the five-year follow-up (*p* ≤ 0.001, Table 2, Figure 6c). At the five-year follow-up, 100 patients (94%) revealed that they would undergo surgery again, while five patients (5%) were not sure, and one patient (1%) would not do the surgery again. The mean satisfaction rate was 9.2 (SD 1.5; R 2–10).

### 3.5. Subgroup Analysis

At the five-year follow-up, Apley’s test was conducted in 72 patients. While 49 patients were able to reach L3 and higher points during Apley’s test at the final follow-up (≥L3 group), 23 patients were not (<L3 group). At baseline, neither group showed statistical differences concerning age, sex, BMI, ROM, subjective and objective outcome scores, or quality of life measurements (Table 3).

While the ≥ L3 group showed a mean height of 168.0 cm, patients of the <L3 group were smaller, with 163.0 cm (*p* = 0.018, Table 3). Concerning the choice of implants, larger glenosphere diameters were chosen in the ≥L3 group (36 mm: n = 11; 39 mm: n = 12; 42 mm: n = 0) than in the <L3 group (36 mm: n = 23; 39 mm: n = 16; 42 mm: n = 10, *p* = 0.044, Table 3). Moreover, the ≥L3 group showed a higher usage of lateralized glenospheres (std: n = 5; +4 mm: n = 44) compared to the <L3 group (std: n = 7; +4 mm: n = 16, *p* = 0.032).

## 4. Discussion

This prospective bicentric case series aimed to evaluate the mid-term outcome of an RSA system using a non-cemented rectangular stem design with metadiaphyseal fixation, in combination with a 135° NSA. The results of this study confirmed already existing satisfactory short-term results in terms of low complication rates and good to excellent clinical as well as radiological results [13], and transferred these into a medium-term follow-up. Moreover subjective mid-term data of this study revealed satisfying results in terms of a 94% rate of patients stating that they would undergo surgery again. These findings are in line with previously published subjective results in short- and long-term follow-ups after RSA, presenting a comparable rate of 94% of patients stating they would undergo this procedure again [2,31].

Melis et al. reported first results concerning radiological changes around the implants, evaluating mid- to long-term results after RSA using a “Grammont style” 155° NSA with a round stem design [32]. The authors found radiolucent lines around the humeral implants at the final follow-up in 57% of all cases. While these authors further reported a higher occurrence of radiolucent lines in cemented stems than in cementless stem designs, our data for a cementless rectangular metadiaphyseal locking stem design revealed 1% (one patient) had signs of incomplete radiolucent lines as well as no signs of implant migration like subsidence, tilt, or shift at mid-term follow-up [32]. Nonetheless, asymptomatic humeral bone resorption was noticed in 3% (two patients) at the metaphysis and in 7% (five patients) at the diaphysis.

While Cuff et al. presented first 5 year data concerning a 135° NSA combined with a round stem design showing a 3% rate of asymptomatic loosening in at least three zones as well as a 9% rate of grade 1 scapular notching, the current data set of a cementless rectangular metadiaphyseal fixation revealed comparable signs of scapular notching but a low rate of asymptomatic humeral loosening [33].

A recent review of 8258 analyzed cases revealed higher rates of scapular notching in 155° Grammont designs compared to all other combined designs (42.5% vs. 12.3%; *p* = < 0.001) [34]. These data are in line with the results of our case series showing a 10% rate of scapular notching at the 5-year follow-up using a 135° NSA combined with a partial inlay design and further support previous biomechanical, CT- based and clinical studies that revealed lower rates of scapular notching and impingement free ROM in terms of adduction by lowering the NSA [11,35,36,37]. Scapular notching is known to result in either scapular bone loss or bone spur formation leading to polyethylene wear and consecutive glenoidal and humeral osteolyses as signs of radiolucency [38,39].

Even though seven patients (10%) of our series revealed signs of scapular notching and eight patients (11%) showed signs of scapular bone spur formation, none of these cases presented either signs of radiolucency or implant-/polyethylene wear. A concomitant lack of implant migration, subsidence, shift, or tilt further leads to the assumption that only high-grade scapular notching (≥grade 2) may cause signs of radiolucency which is supported by data from Mollon et al. showing no sign of radiolucency in patients with grade 2 scapular notching but a 20% rate of radiolucent lines in patients with scapular notching grades 2 and 3 [38].

Despite proven advantages concerning the reduction of scapular notching, lowering the NSA has also biomechanically been theorized to improve rotational capacity [37].

However, Gobezie et al. were not able to show any differences in terms of ROM within a prospective randomized study comparing patients treated with a 135° NSA to patients treated with a 155° NSA (forward flexion: 132° vs. 135°, respectively; *p* = 0.321, external rotation: 29° vs. 30°, respectively; *p* = 0.416) [9].

We further aimed to define pre- and intraoperative factors associated with good or unsatisfactory postoperative internal rotation at the 5-year follow-up. The results of our study showed better internal rotation in the case of usage of lateralized glenospheres as well as larger glenosphere diameters. However, lower patient height was associated with worse internal rotation.

Several previous studies were designed to evaluate factors impacting the ability of postoperative internal rotation after RSA [40,41,42,43,44,45]. While Kim et al. found preoperative internal rotation ability to be the only prognostic factor for postoperative internal rotation [40], studies from Eichinger et al. and Rohman et al. presented different parameters like the Body Mass Index, the preoperative status of the subscapularis tendon, and tobacco usage, as well as the presence of comorbidities to be influencing factors of internal rotation after RSA [43,44]. Therefore, internal rotation seems to be multifactorially affected by patient-specific as well as by implant-/surgeon-specific factors.

In order to objectify positive prognostic factors in ROM following RSA, Boutsiadis et al. published the DSA and LSA. Showing that ranges of the DSA between 40° and 65° and of the LSA between 75° and 90° correlated with increased active elevation and external rotation, these findings are in line with satisfactory results in terms of flexion (142.4°) and external rotation (32°), with a mean DSA of 44.4° (SD 10.9°) and a mean LSA of 86.0° (SD 10.6°) [25].

We observed no cases of instability in the present cohort (0%). Despite using a 135° NSA, recently hypothesized to be a risk factor for instability, the possibility of using a 4 mm lateralized glenosphere that was used in 86% of this cohort (114 patients) may have led to higher joint stability in a prosthetic design (Univers Revers) that was previously described by Werthel et al. to be only moderately lateralizing in comparison to the initial Grammont prosthesis (+7.6 mm global lateral offset) [46].

Further, recently published data focused on different degrees of constraint in currently available RSA designs and their humeral liner. These data revealed consistent results in terms of jump heights, and therefore constant stability ratios and stability trends across different implant sizes [14]. In contrast to implants from other manufacturers with similar NSAs and implant sizes, these consistent results regarding the stability ratio may lead to a high level of interpersonal intraoperative reliability, which may explain the low rate of postoperative instability of 0% observed in the present data set.

This study has some limitations, which can be partly explained by the study’s design. Firstly, even though this case series reports first mid-term results of a new RSA system, it lacks a control group.

Secondly, despite the relatively high follow-up rate regarding PROMs and AEs, the radiographic follow-up was limited to slightly more than half of the patients. This is explained by the length of follow-up and age of the patients, with several patients only willing to report their outcome but not willing to attend a clinical and radiological examination at the hospital.

Further, besides these promising mid-term results concerning ROM as well as complication rates and adverse events, long-term data are needed to prove the consistency of low failure rates and satisfying clinical outcomes.

## 5. Conclusions

This prospective multicentric case series of a reverse shoulder arthroplasty design using a 135° neck–shaft-angle combined with a cementless rectangular metadiaphyseal fixation stem design revealed satisfying, consistent clinical and radiological mid-term results with low complication rates. The combination with glenoid-sided lateralization was associated with improved internal rotation.

## Figures and Tables

**Table 1:** Demographic characteristics of the whole patient cohort, as well as patients followed up 5 years after implantation.

	Original Cohort (n = 132)	5-Year Follow-Up Cohort (n = 105)
Age at surgery (years) *	75 ± 6 (58–91)	75 ± 6 (58–89)
Sex (f/m), n (%)	78 (59)/54 (41)	63 (60)/42 (40)
Body Mass Index (kg/m^2^) *	27 ± 5 (18–42)	27 ± 5 (18–42)
Affected side (left/right), n (%)	45 (34)/87 (66)	38 (36)/67 (64)
Dominant side (left/right/ambidextrous), n (%)	122 (92)/9 (7)/1 (1)	96 (91)/8 (8)/1 (1)
Comorbidities (yes/no), n (%)	118 (89)/14 (11)	91 (87)/14 (13)
Smoking status (yes/no), n (%)	9 (7)/123 (93)	7 (7)/98 (93)

* Continuous baseline characteristics (age, body mass index) are presented as mean values, standard deviation (±SD), and range.

**Table 2:** Subjective and objective outcome parameters.

	Baseline	6-Month FU	1-Year FU	2-Year FU	5-Year FU	*p*-Value
Constant–Murley Score (0–100)	32.9 ± 14.5 (0–75.7)	68.7 ± 10.9 (25.0–97.2)	72.3 ± 11 (36.7–96.2)	72.3 ± 10 (42.3–93.0)	71.7 ± 9.7 (48.2–87.0)	< 0.001
Shoulder Pain and Disability Index (0–100)	38.7 ± 17.1 (4.7–81.4)	82.3 ± 15.0 (14.9–100.0)	86.5 ± 12.8 (36.7–100.0)	85.9 ± 14.7 (31.1–100.0)	86.2 ± 14.0 (31.0–100.0)	<0.001
Subjective Shoulder Value (%)	43.0 ± 17.6 (0.0–80.0)	80.5 ± 14.2 (0.0–100.0)	84.5 ± 11.3 (30.0–100.0)	85.3 ± 10.7 (50.0–100.0)	84.1 ± 13.1 (30.0–100.0)	<0.001
EQ5D5L Utility Index (0–1)	0.62 ± 0.25 (0.06–1.00)	0.90 ± 0.11 (0.41–1.00)	0.91 ± 0.11 (0.38–1.00)	0.90 ± 0.11 (0.40–1.00)	0.87 ± 0.16 (0.28–1.00)	<0.001
EQ5D5L VAS Health Status (%)	69.2 ± 16.5 (25.0–100.0)	81.8 ± 11.3 (40.0–100.0)	83.5 ± 10.1 (50.0–100.0)	82.0 ± 11.1 (40.0–100.0)	79.0 ± 13.0 (45.0–100.0)	<0.001
Flexion (°)	80.0 ± 33.8 (10.0–170.0)	132.1 ± 21.9 (70.0–170.0)	136.6 ± 20.8 (80.0–170.0)	136.9 ± 18.1 (50.0–170.0)	142.4 ± 16.4 (100.0–170.0)	<0.001
Abduction (°)	71.5 ± 28.4 (0.0–170.0)	123.9 ± 23.8 (70.0–160.0)	129.7 ± 22.3 (70.0–60.0)	125.5 ± 19.9 (90.0–160.0)	130.2 ± 21.8 (80.0–170.0)	<0.001
External rotation (°)	32.1 ± 22.4 (0.0–90.0)	29.1 ± 13 (10.0–70.0)	30.3 ± 13.5 (0.0–90.0)	32.4 ± 13.6 (0.0–60.0)	32 ± 14.6 (10.0–80.0)	0.125
External rotation at 90° abduction (°)	20.9 ± 30.2 (0.0–90.0)	59.0 ± 28.1 (0.0–90.0)	64.6 ± 26.4 (0.0–90.0)	57.3 ± 27.3 (0.0–90.0)	52.7 ± 26.6 (10.0–90.0)	<0.001
Maximal						<0.001
internal rotation ability, n (%)						
Lateral aspect of the thigh	8 (8)	2 (2)	1 (1)	0(0)	3 (4)	
Gluteal region	27 (26)	20 (19)	15 (15)	13 (14)	5 (7)	
Lumbosacral region	31 (30)	38 (37)	34 (34)	18 (20)	15 (21)	
L3	21 (20)	27 (26)	31 (31)	29 (32)	27 (38)	
Th12	12 (12)	16 (16)	19 (19)	25 (27)	20 (28)	
Interscapular Th7	4 (4)	0(0)	1 (1)	6 (7)	2 (3)	
Internal rotation at 90° abduction (°)	12.7 ± 18.2 (0.0–70.0)	26.4 ± 16.5 (0.0–80.0)	28.2 ± 14.3 (5.0–70.0)	38.4 ± 14.1 (10.0–70.0)	45 ± 17.8 (6.0–90.0)	< 0.001
Abduction strength (kg)	0.8 ± 1.9 (0.0–12.0)	5.1 ± 2.5 (0.0–15.5	5.7 ± 2.5 (0.0–15.0)	5.6 ± 2.3 (0.0–13.5)	5.2 ± 2.3 (0.0–12.0)	< 0.001

Note: Unless indicated for the maximal internal rotation ability, all other outcome parameters are presented as mean values, standard deviation (±SD), and range.

**Table 3:** Subgroup analyses—association of preoperative parameters with internal rotation ability at the 5-year follow-up.

	Internal Rotation < L3 (n = 23)	Internal Rotation ≥ L3 (n = 49)	*p*-Value
Age at surgery (year) *	75.0 ± 7.0 (62.8–88.6)	74.0 ± 6.0 (58.1–83.5)	0.488
Sex (f/m) n (%)	18.0 (78)/5 (22)	33.0 (67)/16 (33)	0.342
Height (cm) *	163.0 ± 7.0 (147.0–172.0)	168.0 ± 8.0 (156.0–190.0)	0.018
Body Mass Index (kg/m^2^) *	28.3 ± 3.8 (22.1–36.3)	26.7 ± 5.0 (17.7–41.5)	0.179
Constant–Murley Score (0–100) *	28.8 ± 13.7 (0.0–63.9)	32.7 ± 14.9 (9.1–75.7)	0.297
Shoulder Pain and Disability Index (0–100) *	33.8 ± 14.7 (8.3–68.6)	36.2 ± 15.2 (13.1–79.5)	0.528
Subjective Shoulder Value (%) *	38.7 ± 17.6 (0.0–70.0)	44.6 ± 17.7 (10.0–80.0)	0.191
Flexion (°) *	82.0 ± 37.8 (10.0–170.0)	82.4 ± 32.8 (40.0–170.0)	0.964
Abduction (°) *	73.6 ± 36.2 (0.0–170.0)	72.9 ± 27.4 (20.0–170.0)	0.921
External rotation (°) *	33.9 ± 22.2 (0.0–80.0)	28.4 ± 21.4 (0.0–75.0)	0.325
External rotation at 90° abduction (°) *	n = 15 15.3 ± 29.5 (0.0–80.0)	n = 32 19.2 ± 30.3 (0.0–90.0)	0.682
Maximal internal rotation ability, n (%)			0.476
Lateral aspect of the thigh	2 (9)	4 (8)	
Gluteal region	7 (32)	11 (22)	
Lumbosacral region	9 (41)	14 (29)	
L3	3 (14)	9 (18)	
Th12	1 (5)	7 (14)	
Interscapular Th7	-	4 (8)	
Apley’s test of internal rotation (<L3/≥L3), n (%)	18 (82)/4 (18)	29 (59)/20 (41)	0.062
Internal rotation at 90° abduction (°) *	n = 15 7.3 ± 13.2 (0.0–40)	n = 32 13.4 ± 19.7 (0.0–70)	0.282
Abduction strength (kg) *	0.5 ± 1.2 (0.0–4.0)	0.9 ± 2.1 (0.0–12.0)	0.408
Supraspinatus tendon retraction [21], n (%)			0.008
I	-	8 (19)	
II	11 (55)	7 (16)	
III	6 (30)	19 (44)	
n.n.	3 (15)	9 (21)	
Cuff tear arthropathy [29], n (%)			0.207
1	12 (52)	28 (57)	
2	8 (35)	8 (16)	
3	1 (4)	6 (12)	
4A	1 (4)	-	
4B	1 (4)	5 (10)	
5	-	2 (4)	
Glenoid morphology [30], n (%)			0.161
A1	3 (13)	9 (18)	
A2	3 (13)	3 (6)	
B1	1 (4)	-	
B2	-	7 (14)	
B3	-	-	
C	-	2 (4)	
n.n.	16 (70)	28 (57)	
Glenoid morphology [22], n (%)			0.031
E 0	8 (35)	19 (39)	
E I	6 (26)	2 (4)	
E II	-	3 (6)	
E III	-	5 (10)	
n.n.	9 (39)	20 (41)	
Glenosphere size, n (%)			0.044
36 mm	11 (48)	23 (47)	
39 mm	12 (52)	16 (33)	
42 mm	-	10 (20)	
Glenosphere lateralization (std 0 mm/lat + 4 mm), n (%)	7 (30)/16 (70)	5 (10)/44 (90)	0.032

* Unless indicated for categorical parameters presenting absolute and relative frequencies (n, %), all other outcome parameters are presented as mean values, standard deviation (±SD), and range.

## Data Availability

The original contributions presented in this study are included in the article. Further inquiries can be directed to the corresponding author.
